# Asian-Specific total knee system: 5-14 year follow-up study

**DOI:** 10.1186/1471-2474-12-251

**Published:** 2011-11-02

**Authors:** Kunihiro Hosaka, Shu Saito, Takao Ishii, Sei Mori, Takanobu Sumino, Yasuaki Tokuhashi

**Affiliations:** 1 Department of Orthopedic Surgery, Nihon University School of Medicine, 30-1 Oyaguchi, Kamimachi, Itabashi-ku, Tokyo 173-8610, Japan

## Abstract

**Background:**

Knee size and body size differ in Asians compared with Caucasians. Nevertheless, many total knee arthroplasty (TKA) prostheses used worldwide are made for Western Caucasian subjects. As a result, an Asian's knee might not fit these prostheses. We studied the Flexible Nichidai Knee (FNK) system, a new model of TKA for Asian patients. The purpose of this report is to investigate the outcomes of this prosthesis retrospectively.

**Methods:**

We investigated 1055 primary TKAs in 595 patients who underwent FNK for osteoarthritis (OA) in Japan and were followed for > 5 years. The knee score and function score were used for clinical evaluation. We examined the range of motion (ROM) preoperatively and at final follow-up and radiographic assessments. In addition, postoperative complications were investigated. A survivorship analysis was also conducted using two endpoints: revision for any reason and aseptic failure.

**Results:**

890 knees in 502 patients were available for study (follow-up rate of 96.0%). The mean follow-up term was 8.3 years (range, 5.0-14.1 years). The knee and function score significantly improved from 41.3 to 90.3 and from 39.1 to 76.2 points, respectively (p < 0.001). The mean ROM in FNK posterior cruciate retaining (CR) type and FNK posterior-stabilized (PS) type ameliorated significantly from 107.8° and 95.6° to 110.7° and 110.4°, respectively (p < 0.01). Ten knees underwent revision surgery (infection in 3 cases, instability in 2, loosening in 2, and non-union of femoral supracondylar fracture, severe pain, and recurrent hemarthrosis in 1 each). The survivorship rate was 99.4% (95% CI, 99.0-99.8) at 5 years (n = 952 patients at risk) and 96.2% (95% CI, 91.9-100) at 12.5 years (n = 49 patients at risk).

**Conclusion:**

The FNK prosthesis for Asians achieved excellent mid- to long-term survivorship and clinical results.

## Background

Total knee arthroplasty (TKA) is a surgical procedure associated with good success worldwide. In Japan, more than 50,000 patients underwent TKA in 2008, and this number will likely increase as the population ages. However, almost all prosthetic implants have been designed and manufactured to accommodate the knee anatomy of Western Caucasians, and there is some doubt about the application of these systems to Asians, as the femoral size of Asians differs from that of Caucasians [1-8]. Moreover, even if the smallest size from each Western prosthesis company is used, it may be too big for some Asian subjects. Chaichankul and Cheng reported that Thai and Chinese population knees were mismatched and too small, respectively, for several Western prostheses [7,8]. For Japanese subjects, there is no clear evidence about how often incorrect sizing using Western prostheses occurs, but it is likely that results would be similar to other Asian populations. This limitation suggests the need for a prosthesis developed for an Asian population, such as the Japanese [1-8].

**Figure 1:**
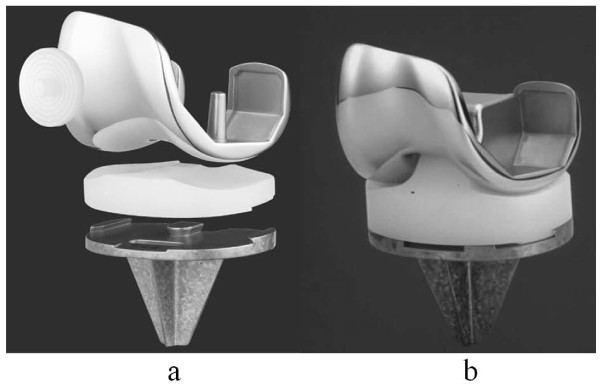
**FNK CR prosthesis and FNK PS prosthesis**. **a**: FNK CR prosthesis; **b**: FNK PS prosthesis.

We have used Flexible Nichidai Knee (FNK) systems (Nakashima Medical Co. Okayama, Japan) (Figure 1), manufactured for Asian patients and designed by Ryu et al., for TKA since July 1995. Moreover, in addition to FNK posterior cruciate retaining (CR) type (Figure 1-a), FNK posterior-stabilized (PS) type (Figure 1-b) has been available since May 1998. Outcomes with these prostheses have not been previously reported. This report describes the outcome at 5 to 14 years follow-up of the FNK system used in Japanese patients with osteoarthritis (OA).

## Methods

Between July 1995 and December 2008, TKAs with the FNK system were implanted in 3143 knees in 1801 patients at Nihon University Itabashi Hospital (Tokyo, Japan). Of those, we investigated a consecutive series of 1055 knees in 595 patients (522 females and 73 males) surgically treated for OA who had undergone TKA between July 1995 and December 2003. All study patients had a minimum of 5 years' follow-up. The preoperative diagnosis was OA including primary disease or secondary disease due to trauma and osteonecrosis in all cases. The mean age of patients at surgery was 72.4 years (range, 41-91 years). Procedures for TKA were performed by six surgeons, including senior authors. All patients provided informed consent to participate in this study.

### Prosthesis

**Figure 2:**
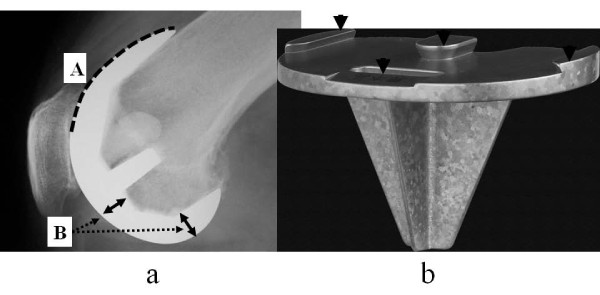
**FNK femoral component and FNK tibial component**. **a**: FNK femoral component. (A) The anterior chamber is thin to decrease pressure on the patellofemoral joint. (B) The standard size (size M) component is 7-mm thick at both the distal and posterior condyles, which is thinner than those in other Western company systems. **b**: FNK tibial component. The FNK tibial component has a 4-point locking mechanism (black arrows), a wide cross-shaped keel to soften the stress in various directions, and is 3.5 mm at the thinnest part to allow retention of original bone.

The FNK implant was developed to fit Asian knees. The femoral component consists of cobalt chromium alloy, and the tibial component is titanium alloy. The design of the FNK system involves a thin anterior chamber and a deep patella groove in the femoral component to reduce pressure on the patellofemoral joint. The femoral component has a multi-radius of rotation in the sagittal plane. Furthermore, the standard size, size M, has a thin, 7-mm component at both the distal and posterior condyles (Figure 2-a). The tibial component has a wide cross-keel to distribute any directional stresses. The thinnest part of the tibial component is maintained at 3.5 mm to keep some of the patient's own bone (Figure 2-b). Designer et al. determined the sizes and shapes of the FNK components based on an original investigation using Japanese cadavers (unpublished data).

The polyethylene insert is manufactured from ultra-high molecular weight polyethylene (UHMWPE) (GUR1020) by direct compression molding from powder, which produces sufficient durability [9] with deep-dish insert shapes. The patellar component consists of all-polyethylene UHMWPE with one peg.

The FNK PS type is 20 mm at the height of the post and 16.8 mm at jumping distance, which offers higher constraints between post-cam mechanisms than other regular PS prostheses and allows the range of ± 2° for varus-valgus constraint, ± 2° at extension position, and ± 5° at flexion position for internal-external rotation constraint. The elasticity load limit from the rear side and lateral to the post is 490N and 580N, respectively (unpublished data). Therefore, the post of the FNK tibial insert is robust enough to bear the stress from the strong constraint.

### Surgical Technique

The skin was cut longitudinally using a lateral, gently curved incision. The capsule was usually opened by midvastus exposure, or medial parapatellar exposure when patellar eversion was expected to be difficult. We generally selected the FNK CR type, but used the FNK PS type in cases with complete disappearance of the anterior cruciate ligament (ACL), potent varus-valgus instability of over 10°, dysfunction of the posterior cruciate ligament (PCL), and fixed flexion contracture of over 15°. In most cases, we chose a cemented implant, but if the patient was younger than 60 years and had high bone quality, a cementless implant was selected. The bone cuts were performed by so-called independent cutting methods. The femur was cut using an intramedullary guide at 7° or 5° of the valgus and to match the transepicondylar axis. The tibia was cut perpendicular to the tibial shaft with a posterior slope of 7° using an extramedullary guide. When severe cartilage defects of the patella or potent pain due to patellofemoral arthritis existed, resurfacing of the patella was performed. A lateral release was undertaken when a lateral patellar shift was observed after implantation. Intraarticular drainage was done for 3 days after surgery. At that point, patients began walking exercises.

From January to December 2009, we investigated all patients by examining them directly, reviewing hospital records, contacting other hospital doctors or practitioners, or interviewing them in detail by telephone or letter for patients who were unable to come to our department.

Clinical evaluation was performed with use of the knee and function scores of the Knee Society clinical rating system [10]. Based on the scores, patient outcomes were classified as excellent (≥ 90), good (80-89), fair (70-79), and poor (< 70). Range of motion (ROM) was measured preoperatively and at final follow-up by the surgeons using a standard goniometer.

For radiological assessment, we examined the transition of the femorotibial angle (FTA) at pre- and post-TKA, existence of radiolucent lines, osteolysis, loosening, patellar dislocation, and polyethylene wear with radiographs.

In addition, we investigated postoperative complications. A survivorship analysis was conducted using two endpoints: revision for any reason and aseptic failure.

### Statistical analysis

Statistical analysis was performed using the Wilcoxon signed-rank test for knee score, function score, ROM, and FTA. A Kaplan-Meier survival analysis [11] was performed with use of revision surgery to remove one or more implants for any reason or for aseptic failure without infection as the end point. This analysis targeted all cases, including deceased and lost patients, whose death or last follow-up date was considered for final assessment. Survivorship outcome included 95% confidence intervals (CI). A p-value ≤ 0.05 indicated statistical significance. All statistical evaluations were performed using SAS software (SAS Institute, Cary, NC, USA).

## Results

Seventy-two patients died during the follow-up period (3 patients had died in the hospital following TKA surgery); 21 patients were lost to follow-up; and 1 patient had undergone revision surgery by the time of death. Ultimately 890 knees in 502 patients were available for study, with a follow-up rate of 96.0%. The mean follow-up period was 8.3 years (range, 5.0-14.1 years). A total of 349 patients were examined directly in our department, and 153 patients were investigated by questionnaire or via information from other doctors. The mean age of follow-up cases was 71.8 years (range 41-91 years) at the time of surgery. Details of TKA surgery for follow-up cases are shown in Table 1.

**Table 1:** Details of TKA surgery for follow-up cases

	No. of patient (N = 502) (%)	No. of knee (N = 890) (%)
**Gender**		
Male	56 (11.2)	97 (10.9)
Female	446 (88.8)	793 (89.1)
**Type of TKA**		
FNK CR		730 (82.0)
FNK PS		160(18.0)
**Methods**		
Simultaneous bilateral	382(76.1)	
Staged bilateral	7 (1.4)	
Unilateral	113 (22.5)	
**Capsule exposure**		
Midvastus exposure		664 (74.6)
Medial parapatellar exposure		226 (25.4)
**Cement or cementless**		
Cement		831 (93.4)
Cementless		59 (6.6)
**Patella component**		
Resurfaced		204 (22.9)
Non-resurfaced		686 (77.1)

### Clinical evaluation (Table 2)

At the last follow-up examination, the mean knee score had significantly improved from 41.3 points (range, 0-87) preoperatively to 90.3 points (range, 47-100) postoperatively (p < 0.001). The preoperative patients were classified by the knee score as follows: good 0.6%, fair 2.2%, and poor 97.2%, and postoperative patients had significantly improved as follows: excellent 64.7%, good 25.4%, fair 6.1%, and poor 3.8%. The function score also indicated a statistically significant improvement from 39.1 points (range, 0-75) preoperatively to 76.2 points (range, 0-100) postoperatively (p < 0.001). The preoperative patients was classified by function score as follows: fair 0.8% and poor 99.2%, and postoperative patients significantly improved as follows: excellent 42.4%, good 20.9%, fair 11.6%, and poor 25.1%. Range of motion was measured in both FNK CR type and PS type. For FNK CR, the mean knee extension and flexion changed from 7.9° (range, -10-50°) and 115.7° (range, 50-145°) before surgery to 0.5° (range, -10-45°) and 111.6° (range, 50-145°) after surgery, respectively, but these differences were not statistically significant. For FNK PS type, the mean knee extension and flexion changed from 10.8° (range, 0-50°) and 106.4° (range, 30-145°) before surgery to 0.6° (range, 0-20°) and 111.0° (range, 70-135°) after surgery, respectively. The mean ROM in FNK CR and PS type also changed significantly from 107.8° and 95.6° to 110.7° and 110.4°, respectively (p < 0.01).

**Table 2:** Clinical evaluation

	Pre-TKA	Final follow-up	p-value
Knee score	41.3 ± 13.5	90.3 ± 8.8	p < 0.001
Class (% of knees)			
Excellent	0.0	64.7	p < 0.001*
Good	0.6	25.4	
Fair	2.2	6.1	
Poor	97.2	3.8	

Function score	39.1 ± 15.2	76.2 ± 27.2	p < 0.001
Class (% of knees)			
Excellent	0.0	42.4	p < 0.001†
Good	0.0	20.9	
Fair	0.8	11.6	
Poor	99.2	25.1	

CR type			
Extension-Flexion (deg.)	7.9-115.7	0.5-111.6	
Range of motion (deg.)	107.8 ± 18.7	110.7 ± 14.1	p < 0.01

PS type			
Extension-Flexion (deg.)	10.8-106.4	0.6-111.0	
Range of motion (deg.)	95.6 ± 25.2	110.4 ± 14.7	p < 0.01

*† Chi-square testing between groups classified by the rating system of knee score and function score.

### Radiographic evaluation

Postoperative radiographs were available in 826 knees (467 patients). A neutral knee axis was defined as an FTA between 170°-175°, a varus knee was defined as an FTA > 175°, and a valgus knee was defined as an FTA < 170° of preoperative FTA. FTA changed from 173.0°, 188.6°, and 161.6° before surgery to 173.4°, 172.9°, and 173.7° after surgery in the neutral axis knee, varus knee, and valgus knee, respectively. The FTA of the varus and valgus knee groups significantly improved compared with pre-operative measurements (p < 0.001 for both). The mean FTA in all post-TKAs was 172.9° ± 2.9. Radiolucent lines were noted in 93 components (5.6%); these components were noted on the femoral side in 27 knees (3.3%) and beneath the tibial component in 66 knees (8.0%). All radiolucent lines had a width of 2 mm or less and did not develop during the course of the study. A radiolucent line on the femoral side in cement and cementless implants was recorded in 15 knees (2.0%) and 12 knees (20.7%), respectively. A radiolucent line beneath the tibial component in cemented and cementless systems occurred in 62 knees (8.1%) and 4 knees (6.9%), respectively. A radiolucent line around tibial screws was seen in 5 knees (8.6%). Asymptomatic osteolysis at the tibia was noted in 25 knees (3.0%). Most osteolysis existed at the inside of the tibia beneath the component, and 1 case progressed to loosening. Loosening, all of which existed on the tibial side, was present in 3 knees (0.4%). Polyethylene wear over 2 mm was noted in 6 knees (0.7%); this was found on X-rays taken with patients standing and in the anteroposterior view; 2 of these knees were the same ones in which loosening was seen. The other 4 cases had no symptoms. A patellar lateralization that showed narrowing outside of the patellofemoral joint space on axial views in X-rays was confirmed in 46 knees (5.6%), but no patient had any symptoms. No knees had a subluxation or a dislocation, which is seen as a partial or a complete disappearance of adaptability of the patella and the femur on axial views in X-rays.

### Complications

Symptomatic pulmonary embolism occurred in 16 cases (3.2%). There were 4 cases (0.5%) with suspected deep infection. A fracture around the component occurred in 7 knees (0.8%), including at the patella in 3 knees, at the supracondylar of the femur in 3 knees, and below the tibial component in 1 knee.

### Survival rate

**Figure 3:**
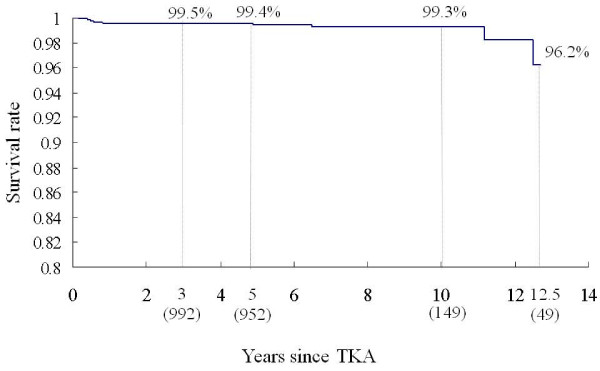
**Kaplan-Meier survivorship using revision for any reason as the end point (n = 1055 knees)**.

**Figure 4:**
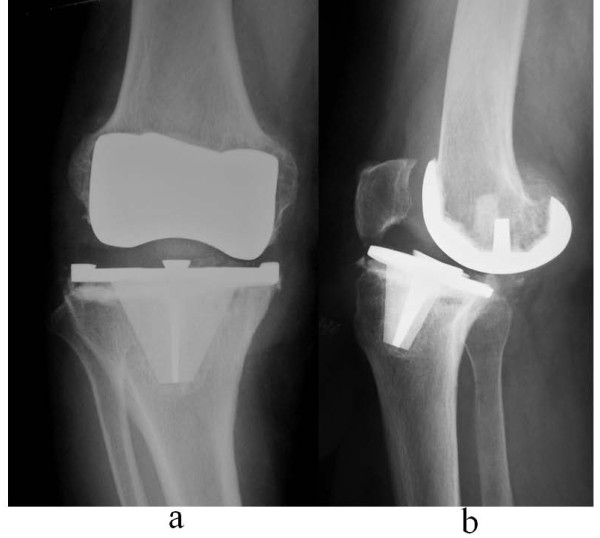
**Aseptic loosening case**. **a**: Anteroposterior view; **b**: Lateral view. Loosening occurred 12.5 years after TKA with the FNK CR type. This patient was female and 70 years old at primary TKA. She underwent a revision of TKA with the FNK PS.

The cumulative survival rate was 99.5% (95% CI, 99.1-99.9) at 3 years (n = 992 patients at risk), 99.4% (95% CI, 99.0-99.8) at 5 years (n = 952 patients at risk), 99.3% (95% CI, 98.7-99.9) at 10 years (n = 149 patients at risk), and 96.2% (95% CI, 91.9-100) at 12.5 years (n = 49 patients at risk) (Figure 3). During the follow-up period, 10 knees underwent revision for any reason (infection in 3 cases, instability in 2, loosening in 2, and non-union of femoral supracondylar fracture, severe pain, and recurrent hemarthrosis in 1 each) (Table 3). Figure 4 shows a case that underwent revision for loosening. The cumulative survival rate without infection was 96.4% (95% CI, 92.1-100) at 12.5 years.

**Table 3:** Revision cases

**No**.	Age	Sex (M/F)	Cause	Time to revision(y)
1	83	F	Instability	0.2

2	54	F	Severe pain	0.4

3	79	F	Infection	0.5

4	72	M	Infection	0.8

5	72	F	Non-union of supracondylar femoral fracture	0.8

6	73	F	Instability	5.0

7	82	M	Recurrent hemarthroses	6.5

8	58	M	Aseptic loosening and polyethylene wear	11.2

9	70	F	Aseptic loosening and polyethylene wear	12.5

10	69	F	Infection	13.7

## Discussion

**Figure 5:**
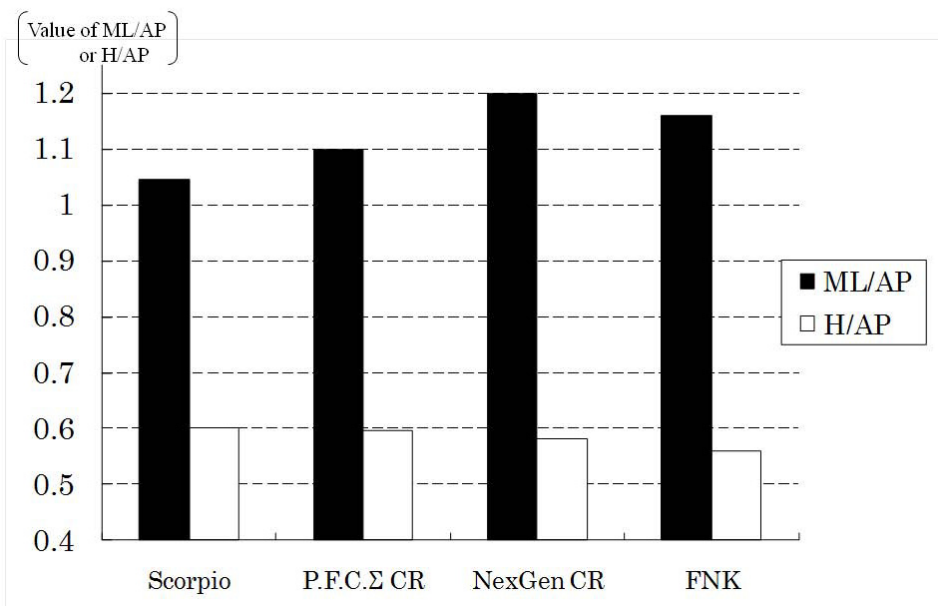
**Comparison of the mediolateral (ML) ratio to anteroposterior (AP) ratio and posterior condylar height (H) to AP height**. This graphic shows the ratio of the size of each model with around 64 mm for ML width of the femur. Scorpio is size 6 (Stryker, Mahwah, NJ, USA), PFC sigma CR is size 2.5, NexGen CR is size C, and FNK is size M.

Distal femoral morphology of Caucasian patients differs from that of Asian populations [1-8]. Urabe et al. showed that the anteroposterior and metaphyseal widths of the anterior and resected condyles were longer in Caucasian women than in Japanese women, but the posterior condyle was longer in Japanese women [3]. Tang et al. reported that the tibial axis was located anterolateral to the center of the tibial plateau in Chinese subjects [1]. Although some data on Japanese populations exist [12,13], the ratio of the mediolateral area to the anteroposterior area in the femoral component of the FNK, which is based on Japanese cadavers, is set close to the mean value of other prostheses, whereas the ratio of posterior condylar height to the anteroposterior area is set at a relatively lower value than other prostheses (Figure 5). Because different Asian populations have similar body measurements, it is likely that this prosthesis will suit other Asian populations in addition to Japanese subjects. The FNK includes 7 mm at both the distal and posterior condyles of the femur compared with 9 mm and 8 mm for all the sizes in PFC sigma (Depuy, Warsaw, IN), and 9 mm and 11 mm in C size from NexGen CR type (Zimmer, Warsaw, IN). In addition, the FNK system preserves as much original bone as possible; this reduces the need for allogenic bone grafts.

The FNK system exhibited both excellent functionality and long durability at mid- to long-term follow-up examinations. The knee and function score were significantly improved compared with before surgery. A movable range was extended in the CR as well as PS type, although the flexion angle of the CR type was not improved. The survival rate of this study was comparable to other reports [14-20]. The reasons for revisions in this series also were similar to those reported in previous studies [21]. Because Japanese people have the longest life expectancy in the world, the good survivorship seen with the FNK may be associated with improved quality of life. Radiolucent lines were noted in 5.6% of cases, but all of them were non-progressive lines with no clinical relevance [22].

FNK PS is a semi-constraining type, which is more similar to the Constrained Condylar Knee type (Zimmer) than regular PS prostheses. Therefore, this model can also correspond to severe deformities and instability without an intramedullary stem. Further, this type of constrained implant has demonstrated a lower rate of patellar complications than other types of prostheses, despite its being characterized by nearly complete constraints [19].

The strength of this report is that it included a large number of patients. The present study, which included several surgeons, one institution, and one implant, represents a common set-up for many patients, thus providing representative data in terms of long-term survivorship as well as clinical outcome [23]. Moreover, this study included only patients with OA. Including only one disease does not necessarily represent a bias. The limitations of the present study are that we did not include any patient-based quality of life evaluations, such as the Short Form-36 or Western Ontario and McMaster Universities Osteoarthritis Index. In addition, we were not able to personally view radiographic findings and ROM for patients who could not come to our hospital. Further, the FNK implant should be compared directly and prospectively with Western implants in an Asian population.

## Conclusion

The FNK prosthesis for Asians resulted in excellent mid- to long-term results in terms of clinical and radiographic evaluations and cumulative success rate in our single institution. Further, there were few complications associated with this prosthesis.

## Competing interests

The authors declare that they have no competing interests.

## Authors' contributions

SS, TI and SM were the senior surgeons for this TKA surgery and performed the clinical investigation. KH carried out the statistical analyses and drafted the manuscript, and TS revised the manuscript. YT was the faculty supervisor of this study. All authors have read and approved the final manuscript.

## Pre-publication history

The pre-publication history for this paper can be accessed here:

http://www.biomedcentral.com/1471-2474/12/251/prepub
